# A Systematic Review of the Pulmonary Microbiome in Patients with Acute Exacerbation COPD Requiring ICU Admission

**DOI:** 10.3390/jcm13020472

**Published:** 2024-01-15

**Authors:** Sjoerd van der Bie, Mark E. Haaksma, Ben Vermin, Hidde van Assema, Eric C. M. van Gorp, Thomas Langerak, Henrik Endeman, Dominic Snijders, Johannes P. C. van den Akker, Marlies A. van Houten, Steven F. L. van Lelyveld, Marco Goeijenbier

**Affiliations:** 1 Department of Intensive Care Medicine, Spaarne Gasthuis Hoofddorp, 2134 TM Hoofddorp, The Netherlands; svanderbie@spaarnegasthuis.nl (S.v.d.B.); mhaaksma@spaarnegasthuis.nl (M.E.H.); bvermin@spaarnegasthuis.nl (B.V.); hvanassema@spaarnegasthuis.nl (H.v.A.); 2 Department of Viroscience, Erasmus MC, 3000 CA Rotterdam, The Netherlands; e.vangorp@erasmusmc.nl (E.C.M.v.G.); t.langerak@erasmusmc.nl (T.L.); 3 Department of Intensive Care Medicine, Erasmus MC, 3000 CA Rotterdam, The Netherlands; h.endeman@erasmusmc.nl (H.E.); j.vandenakker@erasmusmc.nl (J.P.C.v.d.A.); 4 Department of Pulmonology, Spaarne Gasthuis Hoofddorp, 2134 TM Hoofddorp, The Netherlands; dsnijders@spaarnegasthuis.nl; 5 Department of Pediatric Medicine, Spaarne Gasthuis Hoofddorp, 2134 TM Hoofddorp, The Netherlands; MavanHouten@spaarnegasthuis.nl; 6 Department of Internal Medicine, Spaarne Gasthuis Hoofddorp, 2134 TM Hoofddorp, The Netherlands; s.van.lelyveld@spaarnegasthuis.nl

**Keywords:** COPD, bacterial microbiome, ICU, mechanical ventilation

## Abstract

*Background*: Chronic obstructive pulmonary disease (COPD) is a major health concern. Acute exacerbations (AECOPD) may require intensive care unit (ICU) admission and mechanical ventilation. Acute infections and chronic colonization of the respiratory system are known to precipitate AECOPD. Detailed knowledge of the respiratory microbiome could lead to effective treatment and prevention of exacerbations. *Objective*: The aim of this review is to summarize the available evidence on the respiratory microbiome of patients with a severe AECOPD requiring mechanical ventilation and intensive care admission. *Methods*: A systematic literature search was conducted to identify the published papers until January 2023. The collected data were then subjected to qualitative analysis. After the first analysis, a secondary focused review of the most recent publications studying the relationship between microbiome and mortality in AECOPD was performed. *Results*: Out of 120 screened articles six articles were included in this review. Potentially pathogenic microorganisms (PPMs) were identified in 30% to 72% of the patients with community-acquired bacteria, gram-negative enteric bacilli, *Stenotrophomonas* and *Pseudomonas* being the most frequently isolated. During hospitalization, 21% of patients experienced colonization by PPMs. Adequate antimicrobial therapy resulted in the eradication of 77% of the identified PPMs. However, 24% of the bacteria displayed multi-drug resistance leading to prolonged or failure of eradication. *Conclusion*: PPMs are prevalent in a significant proportion of patients experiencing an AECOPD. The most identified PPMs include community-acquired pathogens and gram-negative enteric bacilli. Notably, no differences in mortality or duration of ventilation were observed between patients with and without isolated PPMs. However, the included studies did not investigate the virome of the patients, which may influence the microbiome and the outcome of infection. Therefore, further research is essential to comprehensively investigate the complete microbial and viral composition of the lower respiratory system in COPD patients admitted to the ICU.

## 1. Introduction

Chronic obstructive pulmonary disease (COPD) is a major health concern, ranking as the third leading cause of death worldwide [1]. Acute exacerbations (AECOPD) contribute significantly to this high mortality and may necessitate admission to the intensive care unit (ICU) for mechanical ventilation [2,3]. As such, identifying risk factors for exacerbation is essential.

Acute and chronic respiratory infections play an important role in this regard [4,5]. With this in mind, there has been an increased interest in the microbiome of the lower respiratory tract in patients with COPD. The microbiome is defined as the collection of all microorganisms and their genes in a particular environment [6]. While conventional microbiological culturing once deemed a sample to be sterile, further examination utilizing 16 s ribosomal RNA (rRNA) analysis has revealed the existence of a bacterial microbiome within the lower respiratory tract [7]. 

In individuals diagnosed with COPD, aberrations in immune function and heightened mucus production collectively foster an augmented bacterial burden, leading to the proliferation of potentially pathogenic microorganisms (PPM) [4,8]. This overgrowth of PPMs within the respiratory tract is closely linked to escalated airway inflammation, thereby playing a pivotal role in the progression and exacerbation of the disease [9,10,11,12,13]. Furthermore, a diminished diversity in the microbiome of the respiratory tract has been correlated with prolonged hospitalization and increased mortality rates, underscoring the potential impact of microbiome alterations on the overall disease burden and quality of life for COPD patients [14,15]. Notably, a recent investigation illuminated the association between a dysregulated gut-lung axis, induced by pseudomonas, and unfavorable clinical outcomes in individuals diagnosed with bronchiectasis [16]. Furthermore, several studies showed that an increased gut microbiome diversity and reduced levels of short-chain fatty acids (SCFA) production are associated with disease progression in patients with COPD [17,18,19]. These microbiome-derived metabolites, including SCFAs, as well as long-chain fatty acids and indoles, have the capacity to modulate the host immune response by regulating regulatory T cells and pro-inflammatory factors [20]. This potentially leads to a target for future intervention. Consequently, there has been an increased interest in comprehending the role of the lower respiratory tract microbiome in patients experiencing exacerbations, to improve clinical outcomes and quality of life. 

To date, the majority of research investigating the respiratory system microbiome of patients with COPD has focused on individuals with stable disease or those experiencing non-severe AECOPD who were managed in outpatient clinics or regular wards. Here, it is found that the respiratory microbiome of patients with mild and moderate COPD is not different from healthy controls [7]. However limited data are available on the alterations of the microbiome in patients with a severe exacerbation requiring ICU admission and invasive ventilation, even though their course of disease is more complicated with a higher mortality and increased burden of disease. Therefore, the aim of this systematic review is to summarize the available evidence on the respiratory microbiome of patients with severe AECOPD requiring mechanical ventilation. 

## 2. Materials and Methods

### 2.1. Study Registration and Literature Search

The study was prospectively registered in the open science framework (https://osf.io/gn4uf/, accessed on 4 June 2023). A PubMed (Medline) search was conducted using both keywords and Mesh terms for all studies published until January 2023 (Appendix A). All data extraction and evaluation procedures were performed in accordance with the guidelines outlined in the Preferred Reporting Items for Systematic Reviews and Meta-Analyses (PRISMA) (Appendix B) [21]. 

**Figure 1:**
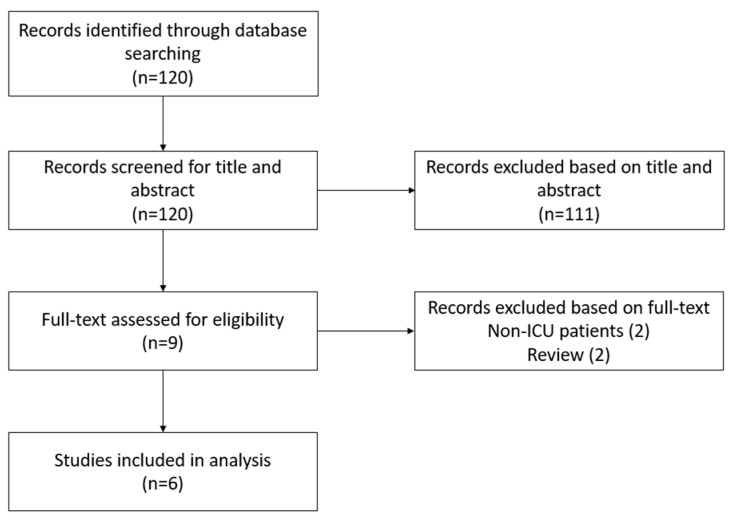
Studies identified in PubMed between January 2010 January 2023.

The initial search identified 120 publications (Figure 1). After screening for title and abstract, 111 articles were excluded. The full text of nine articles was screened for eligibility, of which three articles were excluded. Finally, six articles were included for analysis, consisting of one retrospective cohort and five prospective cohort studies. All the characteristics of the included papers are shown in Table 1. 

### 2.2. Selection Criteria and Data Extraction

The title and abstract of the identified studies were screened by two authors in accordance with predetermined inclusion criteria. In case of a disagreement between the two reviewers, a third reviewer was consulted. In short, studies were eligible if the study populations consisted of patients with severe exacerbation requiring mechanical ventilation and/or admission to the ICU and reported data on microorganisms of the respiratory system. Papers were excluded if the population consisted of children (age < 18 years), stable disease or exacerbations without the necessity for ICU admission, or subjects with another obstructive respiratory disease than COPD. Furthermore, reviews and case reports were excluded. Finally, papers written in another language than English were also excluded. Data extraction was performed independently by one researcher (SB). The following information was extracted from each study: author details, study location, year of publication, study design, in- and exclusion criteria, and outcomes.

### 2.3. Data Collection

Due to the nature of the extracted data, it was not feasible to conduct a quantitative meta-analysis by pooling data. Therefore, a qualitative analysis of the extracted data was used to conduct a systematic review of the relevant literature. Similarly, a formal assessment of data quality was not conducted. 

## 3. Results

### 3.1. Respiratory Microbiome

One of the included studies found a significant number of bacterial taxa in the respiratory system of patients [23]. Bacterial taxa are defined as a group of bacteria sharing at least 97% sequence homology within the 16S rRNA gene sequence. 16s rRNA analyses conducted on tracheal aspirates detected a total of 1213 bacterial taxa in the airway samples obtained during exacerbation [23]. In the case of recent antibiotic pre-treatment, a lower number of taxa (411 (SD 246)) were detected than in patients without pre-treatment; however, there was considerable variability observed between patients [23]. Conversely, another study showed that there was no significant disparity in the quantity of bacterial pathogens found in the lower respiratory tract among patients who had undergone prior antibiotic treatment compared to those who had not received any pre-treatment [24]. Additionally, in patients with fewer taxa, more members of the Pseudomonadaceae were detected, whereas, in patients with a higher bacterial richness, bacterial taxa such as the *Clostridiacae*, *Lachnospiracae*, *Bacillaceae*, and *Peptostreptococcaceae* were more common [23]. Furthermore, this study reported that all patient samples comprised taxa containing species with pathogenic potential, such as *Arcobacter cryaerophilus* and *Brevundimonas diminuta* [23].

#### 3.1.1. Bacterial Species

A PPM refers to a type of microbe that has the inherent capability to cause disease or infection in a host organism [28]. Four studies presented data on the presence of PPMs in their samples [22,24,26,27]. PPMs were isolated in a range of 30% to 72% of the patients [22,24,26,27]. Persistent PPMs after 72 h of antibiotic treatment were reported in 26–32% of the samples, while newly identified PPMs were isolated in 21% [22,24]. However, the initial microbial treatment was deemed inadequate in most of the patients. Table 2 displays the data on PPMs obtained from each individual study.

The two most commonly isolated PPMs were community-acquired bacteria (56–70%) and Gram-negative enteric bacilli (GNEB), *Pseudomonas*, *Stenotrophomonas* spp. (30–44%) [22,24,25]. Table 3 provides a comprehensive list of all the isolated bacteria identified in the included studies.

Important differences were present between invasively and non-invasively ventilated patients. Upon admission, the presence of community-acquired PPMs was found to be more prevalent in the group needing invasive mechanical ventilation (IMV) compared to patients receiving non-invasive ventilation (NIV) [26]. Additionally, during follow-up colonization was more common among patients receiving IMV compared to those undergoing NIV. The most frequently isolated PPMs in this period were nonfermenting gram-negative bacilli (GNB) and GNEB [26]. Notably, colonization by Methicillin-resistant Staphylococcus aureus (MRSA) was most frequent in the subgroup of patients for whom non-invasive ventilation had failed so they needed IMV [26]. 

#### 3.1.2. Serological Analysis

Serological samples were analyzed in two of the included studies [22,24]. A sample was considered positive in the case of seroconversion or a minimum of a four-fold rise in antibody titer. Among the patients, 40% exhibited serological evidence of infection [22,24]. Serology was performed in these for *Chlamydia pneumoniae* (18%), *Influenza virus* (13%), and *Coxiella burnetii*, *Chlamydia psittaci*, and *respiratory syncytial virus*, each identified in one case, accounting for 3% of the population. Sixty percent of patients with *C. pneumoniae* and *influenza* had concomitant PPMs in their tracheal aspirate as well [24]. 

### 3.2. Antimicrobial Therapy

#### 3.2.1. Eradication of Pathogens

Three of the included studies evaluated antibiotic eradication of pathogens [22,24,27]. One of the studies showed that 77% of the isolated PPMs were eradicated when adequate initial antimicrobial therapy was administered [22]. 

Repeated tracheal bronchial aspirate sampling (TBAS) 72 h post start of treatment showed persistent PPMs in 26–32% of the cases [22,24]. All strains of *S. pneumoniae*, *M. Catarrhalis,* and *GNEB* were eradicated. Nonetheless, certain strains of *Pseudomonas* and *Stenotrophomonas* persisted [24]. However, both pathogens were not adequately covered with empirical antimicrobial treatment. Repeated sampling also showed new isolates of GNEB and *P. aeruginosa* in 21% of the patients [22]. These patients were initially treated with amoxicillin-clavulanic acid, ceftriaxone, or cefotaxime. Moreover, five patients (36%) without PPMs in the initial sample had new PPMs (*S. aureus*, *GNEB*, and *Stenotrophomonas*) at the 72 h follow-up sampling [22]. There was no significant association between inadequate initial antimicrobial treatment and the emergence of new PPMs [22].

#### 3.2.2. Antimicrobial Resistance

Antimicrobial resistance was reported by two of the included studies [22,27]. Multidrug-resistant (MDR) bacteria were found in 8% of the patients with a positive tracheal aspirate and accounted for 24% of all the isolated bacteria [27]. Furthermore, a high level of antimicrobial resistance across all isolated strains was found. All strains of *Streptococcus pneumoniae* displayed resistance to penicillin, 50% showed resistance to cotrimoxazole, 40% to cefuroxime and cefotaxime, 60% to erythromycin, and 50% were resistant to imipenem. Of the *Haemophilus influenzae* isolates, 33% exhibited β-lactamase activity. The majority of *Moraxella catarrhalis* isolates (80%) tested positive for β-lactamase [22].

### 3.3. Clinical Outcomes

Several clinical outcomes were evaluated. These included mortality, duration of mechanical ventilation, duration of hospital stay, nosocomial infections, severity of airflow obstruction, acute clinical illness, and NIV success. Table 4 shows the clinical outcomes reported by each study. 

Mortality was significantly higher in patients with MDR-bacteria compared to those infected with other bacterial strains [27]. This association was attributed to inadequate initial antimicrobial treatment rather than the presence of the MDR pathogen itself. The duration of mechanical ventilation and length of stay in the ICU did not show any significant differences between patients with PPMs receiving adequate antimicrobial therapy and those with PPMs receiving inadequate therapy [22]. Furthermore, there was no difference in the duration of mechanical ventilation between patients with eradicated PPMs and persistent PPMs [22,23]. One study reported a correlation in AECOPD patients between the duration of mechanical ventilation and the density of the microbiome, meaning a less rich or dense microbiome was associated with a longer duration of IMV [23]. 

Nosocomial respiratory infections were more prevalent in patients on IMV and patients with NIV failure compared to patients successfully undergoing NIV [26]. Moreover, colonization by nonfermenting GNB was significantly associated with NIV failure [26]. Airway colonization at follow-up and inadequate coverage of antibiotics were significantly associated with hospital mortality. Additionally, usage of antibiotics 48 h before admission was associated with NIV failure [26]. Another study, employing multivariate analysis, indicated that prior antibiotic usage and previous endotracheal intubation were independent risk factors for developing MDR bacteria [27].

#### Mortality

Although this systematic review focused on microbiome alterations and their association with ICU admittance for mechanical ventilation, a significant number of recent studies have been published with a specific focus on mortality. In addition to our systematic search, a focused, non-systematic search from 2015 on microbiome-associated mortality in patients with AECOPD without ICU admission was performed. This search identified an additional four studies on mortality and the microbiome in patients with COPD [14,29,30,31]. Two studies demonstrated that decreased microbial diversity, the absence of *Veillonella*, and an abundance of *Staphylococcus* were associated with increased mortality [29,31]. Moreover, a higher abundance of *Proteobacteria* appears to be linked to an increased neutrophil count and mortality [14,30]. Additionally, the proportion of Veillonella seems to decrease in patients with more frequent exacerbations, while the proportion of *Staphylococcus* increases [32]. 

## 4. Discussion

This systematic review provides a summary of the literature on the microbiome of the respiratory tract in ICU-admitted patients due to severe exacerbation of COPD requiring mechanical ventilation. A major finding was the restricted availability of data on the bacterial microbiome and especially virome from patients undergoing IMV for AECOPD. There are four key findings to consider. First, several studies demonstrated the presence and effective eradication of PPMs in a large part of the studied population. Second, a high incidence of MDR bacteria was reported in several studies. Third, prior and inadequate anti-microbial therapy as well as MDR bacteria were linked to an elevated risk of mortality. Fourth, during admission, community-acquired PPMs were more prevalent in patients necessitating IMV compared to patients receiving NIV. 

Previous studies have documented the quantity and type of PPMs in individuals with an exacerbation of COPD not necessitating ICU admission [33,34,35]. In those studies, the quantity of PPMs isolated seems to be comparable to patients who do need admission to the ICU. Regarding species, *H. influenzae, M. catarrhalis, S. pneumoniae, P. aeruginosa*, and *S. aureus* were most frequently isolated [12,34,35,36,37]. These findings are also consistent with the results of the included studies in this review. The only notable difference was a high incidence of *C. pneumoniae* and *S. oralis*. 

This suggests that there is no disparity in the bacterial pathogens accountable for exacerbations between patients who are admitted to an ICU and those who are not. However, explaining alterations in the microbiome during exacerbation poses a challenge. These changes could be attributed to microbiome alterations, but acute infection might also play a significant role. Furthermore, other factors contributing to a severe exacerbation should be considered.

An important consideration is that the current evidence, this review included, only entails the characterization of the bacteriome. Differences between the respiratory tract virome, i.e., all viruses, including bacteriophages, that are present in the respiratory tract, have not yet been investigated but could play an important role as well. Indeed, a previous study showed that the spectrum of respiratory viruses differed between patients with COPD and asthma during exacerbation, indicating that disease-specific factors may be responsible for susceptibility to certain viruses [38]. Furthermore, the microbiome of a patient with AECOPD undergoes changes during exacerbations triggered by viral infections [7]. Moreover, non-pathogenic viruses, particularly anelloviruses, have been investigated in lung transplant patients and are linked to primary graft dysfunction [39,40]. Their role, and those of other viruses, such as bacteriophages, remains to be elucidated in patients with COPD requiring ICU admission.

In addition, oral and inhaled corticosteroids appear to influence the respiratory microbiome and alter the course of the disease. Treatment with corticosteroids is common in patients with advanced COPD. Previous research suggests that the use of corticosteroids results in an increased bacterial load in sputum [41]. Furthermore, steroids lead to a reduction in microbiome diversity and an elevation in the Proteobacteria-to-Firmicutes ratio [41,42,43]. While these factors could potentially impact the microbiome and, consequently, the course of the disease, further research is essential. 

Importantly, four of the six included studies excluded patients with radiographically confirmed bronchiectasis. While patients with COPD and bronchiectasis seem to have clinically a more advanced severity of disease [44]. This exclusion might influence the data of patients with severe COPD. While both conditions often co-exist and can lead to exacerbations, it has been demonstrated that bronchiectasis influences the microbiome on its own towards a lower diversity [45,46,47,48]. The most commonly isolated pathogens in bronchiectasis are *Haemophilus influenzae, Pseudomonas aeruginosa, Stenotrophomonas,* and *Non-tuberculous Mycobacteria (NTM)* [47,49]. Furthermore, a recent cohort study demonstrated that during exacerbation *Acinetobacter baumannii, Mycobacterium tuberculosis, Haemophilus influenzae, Haemophilus parahaemolyticus, Abiotrophia defectiva,* and *Miomonas micros* were significantly more present [48]. These findings differ from the studies included in this review involving patients with AECOPD but without bronchiectasis. By excluding patients with bronchiectasis, there is a risk of missing crucial differences in the microbiome among patients with severe COPD. The anatomical changes and more frequent use of antibiotics in patients with bronchiectasis could potentially influence their microbiome, thereby complicating the course of the disease

High rates of bacterial resistance in the lower respiratory tract have been previously documented in patients with acute exacerbation, regardless of the necessity for ICU admission. Several strains of *A. baumanii* and *S. pneumoniae* showed intermediate to high-level resistance [50,51]. Moreover, 33% of the *H. influenzae* and 95% of the *M. catarrhalis* strains demonstrated β-lactamase activity [52]. This implies that the quantity of MDR bacteria is similar between ICU and non-ICU patients. Consequently, it seems unlikely that this influences the severity of exacerbation. Nevertheless, this reiterates the importance of antibiotic stewardship and fast adequate treatment in this patient group. The findings demonstrated that inadequate antibiotic treatment, a common occurrence in patients carrying MDR bacteria, could lead to increased mortality rates and prolonged mechanical ventilation durations, as similarly documented in prior studies [35,53]. Initial inadequate antibiotic treatment, more commonly in patients with MDR bacteria, may lead to persistent infection and increased severity of exacerbation necessitating ICU admission. Furthermore, other complicating factors in patients with MDR should be considered. The elevated levels of MDR bacteria may result from more frequent antibiotic usage, indicating a potentially more severe form of COPD in the first place. These factors could influence poorer clinical outcomes as well. 

Furthermore, the included studies suggest that most PPMs can be effectively eliminated through appropriate antimicrobial therapy. However, patients who are intubated face an elevated susceptibility to lower respiratory tract colonization, which can lead to the emergence of new PPMs or bacterial overgrowth, subsequently complicating ongoing treatment or even increasing mortality [54,55]. In patients undergoing NIV, the incidence of nosocomial respiratory infections and colonization by pathogens was comparatively lower. However, the presence of nonfermenting GNB colonization in NIV patients was significantly associated with NIV failure and the subsequent requirement for IMV. This observation implies that colonization with GNB may contribute to increased disease severity and mortality. Additionally, there were no significant differences in the duration of ventilation, mortality rate, and duration of ICU admission between patients with or without PPMs. This suggests that the identification of specific pathogens may not have direct clinical relevance for the treatment of these patients. However, the strict inclusion criteria of this review might influence the clinical outcomes of this study, by leading to a small patient population. Previous studies showed that an absence of *Veillonella* and an abundance of *Staphylococcus* was associated with mortality [29,31,32]. Furthermore, it is important to note that most of the included studies relied on conventional culturing methods for pathogen isolation. It is possible that some analyses yielded false negative results, and the relevant pathogen may not have been accurately identified. Additionally, it is worth considering that other factors such as the previously mentioned virome, may also contribute to the disease progression. Future research focusing on the analysis of the microbiome and virome in patients with AECOPD is essential to gain a deeper understanding of the underlying mechanisms involved.

We acknowledge certain limitations. The research conducted on the microbiome of patients experiencing an AECOPD primarily encompasses non-ICU patients. Consequently, our review is constrained by the scarcity of available studies on this topic. Therefore, the number of patients included in our analysis is relatively small, which introduces the potential for selection and publication bias. Additionally, the limited geographical distribution of the included studies in this review restricts the extrapolation of the findings. Furthermore, the majority of the studies included in our analysis employed traditional culturing techniques to isolate bacteria, rather than utilizing 16S rRNA analysis. This leads to a lower sensitivity for identifying bacterial species, thereby providing a limited representation of the complete bacterial microbiome. However, it is important to highlight that the included studies did evaluate the most prevalent pathogenic bacteria, which likely contributed to disease manifestation in most cases. Additionally, the diversity in reported outcomes in the included studies could be a limitation of this review. The majority of the studies primarily emphasized the prevalence of PPMs. As a result, there are less data available about alternative bacterial species, antimicrobial resistance, and eradication of pathogens. Nevertheless, to the best of our knowledge, this represents the first systematic review examining the respiratory microbiome of patients with COPD necessitating admission to the ICU and/or mechanical ventilation.

## 5. Conclusions

In conclusion, during ICU admission caused by exacerbation COPD, PPMs were identified in the majority of patients, primarily comprising community-acquired pathogens and GNEB. No significant disparities in mortality and duration of ventilation were observed between patients with and without PPMs. However, these studies did not investigate the virome or the influence of viruses on the microbiome and the pathogenesis of exacerbation.

Further research is essential to evaluate the microbiome, and other factors such as the virome, of the lower respiratory tract in ICU-admitted COPD patients to gain a deeper understanding of the underlying mechanisms. Preferably in a prospective study design. 

## Figures and Tables

**Table 1:** Characteristics of included studies.

Author	Year	Study Type	Sample Size	Inclusion Criteria	Exclusion Criteria	Study Design	Outcomes
Ewig et al., Spain [22]	2000	Prospective cohort	50	Diagnosed with an exacerbation COPD	Clinical or radiographic evidence of bronchiectasis	TBAS, PSB, and BAL within 24 h of mechanical ventilation	PPM
				Severe respiratory failure requiring mechanical ventilation	Infiltrates on chest radiograph	Sampling repeated after 72 h	Anti-microbial resistance patterns
				No hospital admission last 3 months before trial	Severe immunosuppression, malignancies, and coagulopathies	Paired blood serum samples	Clinical outcomes related to PPM
				No prior antimicrobial treatment within 4 weeks before admission			
Huang et al., USA [23]	2010	Retrospective cohort	8	Diagnosed with an exacerbation COPD	Not specified	Retrospectively 16S rRNA analysis of BAL samples	Bacterial taxa in aspirate
				Mechanically ventilated patients			Clinical outcome related to PPM
				enrolled in parent study of *Pseudomonas aeruginosa* in intubated patients			
Soler et al., Spain [24]	1998	Prospective cohort	50	Diagnosed with an exacerbation COPD	Clinical radiographic evidence of bronchiectasis	Pharyngeal swab, TBAS, BAL within 24 h of mechanical ventilation	PPM
				Mechanical ventilation for hypercapnic respiratory failure	Severe immunosuppression, malignancies, and coagulopathies	Sampling repeated after 72 h	Clinical outcomes related to PPM
				No hospital admission last 3 months before trial		Paired blood serum samples	Anti-microbial resistance patterns
				No prior antimicrobial treatment within 4 weeks before admission			
Tan et al., China [25]	2014	Prospective cohort	53	Acute exacerbation COPD requiring mechanical ventilation	Mild and moderate COPD	Dental plaque and TBAS on first day of admission to ICU	Comparison of bacterial species in plaques and tracheal aspirate
				*S. pneumoniae*, *P. aeruginosa* or *K. pneumoniae* in tracheal aspirate.	Pregnancy or breast feeding		Pathogenic bacterial load
					Periodontal therapy or antibiotics in last 3 months prior to trial		
					Antibiotic treatment in last 3 months prior to trial		
Ferrer et al., Spain [26]	2005	Prospective cohort	137	Clinical symptoms of exacerbation of COPD	Pneumonia and other causes of pulmonary infiltrates	Sputum of patients undergoing NIV within 24 h and after 3 days	PPM
					Prior exacerbation of hospitalization in the previous 2 months	TBAS of intubated patients within 24 h and after 3 days	NIV success rate related to PPM
					Prior hospital stay longer than 24 h during current admission		
					Tracheotomy		
Nseir et al., France [27]	2006	Prospective cohort	857	Acute exacerbation COPD requiring mechanical ventilation > 48 h	Hospitalization > 24 h prior to intubation	Tracheal aspirates at admission to ICU	MDR
					Patients intubated > 24 h		Clinical outcomes related to MDR
					Evidence of bronchiectasis		Risk factors for MDR bacteria

Table 1 shows the study characteristics of the included studies. Abbreviations: TBAS, tracheal bronchial aspirate sampling; PSB, protected specimen brush; BAL, bronchial alveolar lavage; PPM, potentially pathogenic microorganism; MDR, multi-drug resistant.

**Table 2:** Prevalence of PPMs.

Author	PPMs (%)
Ewig et al., 2000 [22]	56
Soler et al., 1998 [24]	72
Nseir et al., 2006 [27]	30
Ferrer et al., 2005 [26]	69 *

Table 2 shows the prevalence of potentially pathogenic microorganisms (PPMs) categorized according to the respective studies. * Subgroup of mechanically ventilated patients.

**Table 3:** List of pathogens isolated in the respiratory tract.

	Prevalence of Pathogens Categorized Per Study (%)				
Bacterial Species	Ewig et al. [22]	Soler et al. [24]	Tan et al. * [25]	Ferrer et al. ** [26]	Nseir et al. [27]
*Acetobacter europaeus*			2		
*Acinetobacter baumannii*			5	0	9
*Aggregatibacter actinomycetemcomitans*			3		
*Arabidopsis thaliana*			1		
*Bacillus subtilis*			1		
*Candida* spp.		6			
*Capnocytophaga sputigena*			6		
*Chlamydia pneumoniae*	13				
*Chlamydia psittaci*	2				
*Chryseobacterium meningosepticum*			3		
*Corynebacterium* spp.		3			
*Coxiella burnetii*	2				
*Enterobacter cloacae*	4	3		6	1
*Enterococcus faecalis*				8	
*Escherichia coli*	2	0		4	1
*Haemophilus influenzae*	23	17	3	21	17
*Klebsiella pneumoniae*			8		1
*Moraxella catarrhalis*	9	6		4	9
*Morganella morganii*					0.9
*Neisseria* spp.		2			
*Peptostreptococcus*			10		
*Porphyromonas gingivalis*			7		
*Proteus mirabilis*	2	2		4	3
*Pseudomonas aeruginosa*	13	14	8	16	10
*Pseudomonas fluorescens*	4	-			
*Saccharomyces cerevisiae*			2		
*Serratia marcescens*	2	2	3		2
*Staphylococcus aureus*			3	2	9
*Methicillin resistant staphylococcus aureus (MRSA)*				4	6
*Staphylococcus epidermidis*		13			
*Stenotrophomonas maltophilia*	4	3			3
*Streptococcus group F*		2			
*Streptococcus mitis*		2			
*Streptococcus oralis*			14		
*Streptococcus pneumoniae*	9	6	12	16	20
*Streptococcus vividans*		20			
*Tannerella forsythis*			4		
*Treponema denticola*			6		
Total bacteria isolated in tracheal samples	53	64	289	51	304

Table 3 presents the prevalence of distinct bacterial species in the respiratory samples of patients, classified based on the corresponding studies that documented the presence of specific bacteria. * Reported in this table are only the isolated bacteria of the tracheal aspirate, percentage compared to total cultured bacteria in tracheal aspirate. ** Subgroup of mechanically ventilated patients.

**Table 4:** Clinical outcomes.

Author	PPM (%)	Study Group	Duration of Mechanical Ventilation (Days (SD))	Duration of ICU Stay (Days (SD))	Nosocomial Infection (%)	Mortality (%)
Ewig et al., 2000 [22]	56	PPM with appropriate antibiotics	7.6 (7.6)	9.4 (7.1)	6	6
		PPM with inappropriate antibiotics	6.4 (4.8)	8.3 (4.9)		
Soler et al., 1998 [24]	72	With PPM	7.4 (6.7)	9.2 (6.7)	6	6
		Without PPM	9.6 (6.5)	10.9 (6.5)		
Nseir et al., 2006 [27]	30	With PPM	10 (11)	15 (14)		30
		Without PPM	7 (9)	12 (11)		24
Ferrer et al., 2005 [26]	69 *	IMV	8.1 (7.2)	10.1 (7.9)	22	18
		NIV-failure	2.2 (1.0)	13.6 (10.7)	41	32

Table 4 presents the clinical outcomes, classified based on the corresponding studies. Abbreviations: PPM, potentially pathogenic microorganism; IMV, invasive mechanical ventilation; NIV-failure, non-invasive ventilation failure requiring mechanical ventilation. * Subgroup of mechanically ventilated patients.

## Appendix A

### Appendix A.1. Search Query

(“Pulmonary Disease, Chronic Obstructive”, “Chronic Obstructive Pulmonary Disease”, ”COPD”, “Chronic obstructive lung disease”) and (“exacerbation”, “Symptom Flare Up”, “symptom flare up”) and (“Microbiota”, ”Microbiota”, “Microbiome”, “microbial”, “colonisation”, “colonization”) and (“Intensive Care Units”, “Intensive Care Unit”, “Critical Care”, “critical care”, “critical illness”, “Critical Illness”, “ICU”, “Respiratory Care Units”, “Respiratory Care Units”, “emergency department”, “Emergency Service, Hospital”, “emergency room”).

## Appendix B

**Table A1:** Prisma guidelines.

Section and Topic	Item #	Checklist Item	Location Where Item Is Reported
**TITLE**			
Title	1	Identify the report as a systematic review.	
**ABSTRACT**			
Abstract	2	See the PRISMA 2020 for Abstracts checklist.	
**INTRODUCTION**			
Rationale	3	Describe the rationale for the review in the context of existing knowledge.	
Objectives	4	Provide an explicit statement of the objective(s) or question(s) the review addresses.	
**METHODS**			
Eligibility criteria	5	Specify the inclusion and exclusion criteria for the review and how studies were grouped for the syntheses.	
Information sources	6	Specify all databases, registers, websites, organizations, reference lists, and other sources searched or consulted to identify studies. Specify the date when each source was last searched or consulted.	
Search strategy	7	Present the full search strategies for all databases, registers, and websites, including any filters and limits used.	
Selection process	8	Specify the methods used to decide whether a study met the inclusion criteria of the review, including how many reviewers screened each record and each report retrieved, whether they worked independently, and if applicable, details of automation tools used in the process.	
Data collection process	9	Specify the methods used to collect data from reports, including how many reviewers collected data from each report, whether they worked independently, any processes for obtaining or confirming data from study investigators, and if applicable, details of automation tools used in the process.	
Data items	10a	List and define all outcomes for which data were sought. Specify whether all results that were compatible with each outcome domain in each study were sought (e.g., for all measures, time points, analyses), and if not, the methods used to decide which results to collect.	
	10b	List and define all other variables for which data were sought (e.g., participant and intervention characteristics, funding sources). Describe any assumptions made about any missing or unclear information.	
Study risk of bias assessment	11	Specify the methods used to assess the risk of bias in the included studies, including details of the tool(s) used, how many reviewers assessed each study, and whether they worked independently, and if applicable, details of automation tools used in the process.	
Effect measures	12	Specify for each outcome the effect measure(s) (e.g., risk ratio, mean difference) used in the synthesis or presentation of results.	
Synthesis methods	13a	Describe the processes used to decide which studies were eligible for each synthesis (e.g., tabulating the study intervention characteristics and comparing against the planned groups for each synthesis (item #5)).	
	13b	Describe any methods required to prepare the data for presentation or synthesis, such as handling of missing summary statistics, or data conversions.	
	13c	Describe any methods used to tabulate or visually display the results of individual studies and syntheses.	
	13d	Describe any methods used to synthesize results and provide a rationale for the choice(s). If meta-analysis was performed, describe the model(s), method(s) to identify the presence and extent of statistical heterogeneity, and software package(s) used.	
	13e	Describe any methods used to explore possible causes of heterogeneity among study results (e.g., subgroup analysis, meta-regression).	
	13f	Describe any sensitivity analyses conducted to assess the robustness of the synthesized results.	
Reporting bias assessment	14	Describe any methods used to assess the risk of bias due to missing results in a synthesis (arising from reporting biases).	
Certainty assessment	15	Describe any methods used to assess certainty (or confidence) in the body of evidence for an outcome.	
**RESULTS**			
Study selection	16a	Describe the results of the search and selection process, from the number of records identified in the search to the number of studies included in the review, ideally using a flow diagram.	
	16b	Cite studies that might appear to meet the inclusion criteria, but which were excluded, and explain why they were excluded.	
Study characteristics	17	Cite each included study and present its characteristics.	
Risk of bias in studies	18	Present assessments of risk of bias for each included study.	
Results of individual studies	19	For all outcomes, present, for each study: (a) summary statistics for each group (where appropriate) and (b) an effect estimate and its precision (e.g., confidence/credible interval), ideally using structured tables or plots.	
Results of syntheses	20a	For each synthesis, briefly summarize the characteristics and risk of bias among contributing studies.	
	20b	Present results of all statistical syntheses conducted. If meta-analysis was conducted, present for each the summary estimate and its precision (e.g., confidence/credible interval) and measures of statistical heterogeneity. If comparing groups, describe the direction of the effect.	
	20c	Present results of all investigations of possible causes of heterogeneity among study results.	
	20d	Present results of all sensitivity analyses conducted to assess the robustness of the synthesized results.	
Reporting biases	21	Present assessments of risk of bias due to missing results (arising from reporting biases) for each synthesis assessed.	
Certainty of evidence	22	Present assessments of certainty (or confidence) in the body of evidence for each outcome assessed.	
**DISCUSSION**			
Discussion	23a	Provide a general interpretation of the results in the context of other evidence.	
	23b	Discuss any limitations of the evidence included in the review.	
	23c	Discuss any limitations of the review processes used.	
	23d	Discuss the implications of the results for practice, policy, and future research.	
**OTHER INFORMATION**			
Registration and protocol	24a	Provide registration information for the review, including the register name and registration number, or state that the review was not registered.	
	24b	Indicate where the review protocol can be accessed, or state that a protocol was not prepared.	
	24c	Describe and explain any amendments to information provided at registration or in the protocol.	
Support	25	Describe sources of financial or non-financial support for the review, and the role of the funders or sponsors in the review.	
Competing interests	26	Declare any competing interests of review authors.	
Availability of data, code and other materials	27	Report which of the following are publicly available and where they can be found: template data collection forms; data extracted from included studies; data used for all analyses; analytic code; any other materials used in the review.	
